# A Novel Method to Handle the Effect of Uneven Sampling Effort in Biodiversity Databases

**DOI:** 10.1371/journal.pone.0052786

**Published:** 2013-01-11

**Authors:** Iker Pardo, María P. Pata, Daniel Gómez, María B. García

**Affiliations:** 1 Conservation of Biodiversity, Pyrenean Institute of Ecology (CSIC), Zaragoza, Spain; 2 Conservation of Biodiversity, Pyrenean Institute of Ecology (CSIC), Jaca, Spain; University of Kent, United Kingdom

## Abstract

How reliable are results on spatial distribution of biodiversity based on databases? Many studies have evidenced the uncertainty related to this kind of analysis due to sampling effort bias and the need for its quantification. Despite that a number of methods are available for that, little is known about their statistical limitations and discrimination capability, which could seriously constrain their use. We assess for the first time the discrimination capacity of two widely used methods and a proposed new one (FIDEGAM), all based on species accumulation curves, under different scenarios of sampling exhaustiveness using Receiver Operating Characteristic (ROC) analyses. Additionally, we examine to what extent the output of each method represents the sampling completeness in a simulated scenario where the true species richness is known. Finally, we apply FIDEGAM to a real situation and explore the spatial patterns of plant diversity in a National Park. FIDEGAM showed an excellent discrimination capability to distinguish between well and poorly sampled areas regardless of sampling exhaustiveness, whereas the other methods failed. Accordingly, FIDEGAM values were strongly correlated with the true percentage of species detected in a simulated scenario, whereas sampling completeness estimated with other methods showed no relationship due to null discrimination capability. Quantifying sampling effort is necessary to account for the uncertainty in biodiversity analyses, however, not all proposed methods are equally reliable. Our comparative analysis demonstrated that FIDEGAM was the most accurate discriminator method in all scenarios of sampling exhaustiveness, and therefore, it can be efficiently applied to most databases in order to enhance the reliability of biodiversity analyses.

## Introduction

Decisions on biodiversity conservation are typically dependent on the degree of knowledge of species distribution [1], therefore, they ideally require the best available spatially explicit information of species distribution [2]. Given that field work necessary to get a database representative of the real biodiversity in large areas is highly resource-consuming, and current funding for this task is scarce [3]–[4], historical data stored in herbaria, museums, atlas and unpublished material emerge as an outstanding alternative [5]. In fact, biodiversity databases compiling information from these sources have proliferated worldwide in the last decade [6], as it is exemplified by initiatives such as the Global Biodiversity Information Facility (GBIF) [http://www.gbif.org]. Scientists and managers can now take advantage of the enormous effort done during decades of biodiversity inventories [7] and raise new ecological questions [6], [8]. In particular, biodiversity databases are being intensively used in relevant conservation issues, such as the predictive distributions of plants and animals under global change scenarios [9]–[10], the identification of biological hotspots (e.g. [11]–[13]), or the design of protected areas [1], [14]. The generation of new analytical tools is promoting advances in the study of these fields, however, their reliability remains challenging due to the contingencies of the baseline data [15]–[22]. For instances, biodiversity database usually contain incomplete distribution data, because the information was collected according to different aims [15]. Evidences of how bias in database information can compromise biodiversity analyses and conservation planning are reported in a large number of studies [19], [20], [23], [24], [25], [26], [27]. Hence, an adequate control of data-quality is needed [15].

Quality control process should regard both database configuration and the evaluation of data suitability for analyses. According to the scheme proposed in Hortal et al. [15], the control routine has two main levels: (i) data-compilation and digitalization, and (ii) sampling effort assessment. The former is related to the reliability of the sources of information, taxonomic bias and the geographical accuracy of the data [8], [28], [29]. The latter refers to the uneven sampling effort present in non-systematic biodiversity databases [30]. As the first control level has already been discussed elsewhere, in this paper we will focus only on the assessment of the sampling effort bias.

Sampling effort is likely to be temporally, spatially and environmentally biased [20], [27], [31], [32], [33]. Temporal bias can be minimized by limiting database information to a time period short enough as to ensure that information remains the same throughout this period. Regarding spatial and environmental bias, it is well known that some territorial units accumulate more sampling records than others due to diverse factors such as accessibility, habitat singularity, abundance of rare taxa, or differences in funding [24], [25], [34], [35], [36], [37], [38]. Given that most aspects of biodiversity (e.g. species richness) correlate well with sampling effort (e.g. [15]), diversity distribution inferred from raw database information may reflect the spatial distribution of sampling effort rather than the real distribution of diversity [8], [15], [27]. Hence, biodiversity distribution analyses based on spatially explicit data should account for sampling effort.

Among the range of methods that have been proposed to reduce the bias of sampling effort, those based on species accumulation curves (SAC) [34], [39], [40] are commonly used. According to SAC's properties, the total number of species recorded rises towards a ceiling as sampling effort increases [39], [41]. Once the SAC is constructed, a model is fitted to describe the accumulative-sampling effort relationship (e.g. [42]). The selection of the model should be done with statistical rigor [39], but also according to the discrimination capability, i.e. the probability of correctly identifying well (or poorly) sampled units [43]. The discrimination is a *sine qua non* criterion in scientific fields with important social responsibility such as clinical diagnostic [44]–[45], whereas it has been hardly applied in ecological classification analysis, including the evaluation of sampling effort. If the methods to assess sampling effort fail to discriminate well from poorly sampled units, the resultant classification would be seriously affected. Likewise, if the discrimination capability differ among methods, then, the reliability of the classification would depend to a great extent on the selected method, and so will do the uncertainty of any analysis based on such information.

In this study, we analyze for the first time the discrimination capability of commonly used SAC based methods to quantify sampling completeness, and present a novel approach. We first compare methods according to their discrimination capability in two contrasting scenarios of sampling exhaustiveness and in an ideal situation, where the true species richness is known. Finally, we define an objective and generalizable procedure to account for sampling effort bias in biodiversity databases using the novel method and discuss its practical benefits for conservation management.

## Methods

### Review of methods to assess sampling effort bias

The SAC are constructed by plotting the expected (mean) cumulative number of species *S(n)*, at a given number of samples (*n*) [40], [46]. Samples order is randomized by repeatedly re-sampling (without replacement) to rule out its effect on the SAC [40], [41], [46]. Two main procedures based on SAC have been proposed to assess the sampling completeness: (i) the proportion of species richness out of the total predicted by the richness estimators [47]–[49], and (ii) the slope of the accumulation curve [30], [50].

For the former procedure, the predicted richness should be calculated first, which can be done in several ways. Extrapolation of SAC based on asymptotic functions is one of them. The predicted richness is estimated as the total number of species that would be achieved with a hypothetical infinite sampling effort. The most usual models used to describe the SAC are the negative exponential, the Clench, and the Weibull models [39], [50], [51], [52], [53]. The other common way of predicting species richness is by non-parametric estimators based on the number of rare species observed within samples, either from incidence or abundance data [54]–[55]. The most common estimators in this case are Chao [56], Jacknife (onwards NPE) and Bootstrap [57] estimators, as well as incidence-based and abundance-based coverage estimators, called ICE and ACE respectively [58]. For a complete review of all these methods see [40], [59] and [60].

The second procedure for measuring sampling completeness is the slope of the SAC along the sampling effort gradient, which is minimum when all species have been found [30], [50]. There are several alternatives to compute the slope of the curve. One is the geometric definition of the slope as the secant line to the curve:
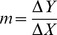
(1)where *Y* is the *s*pecies richness and *X* the measure of effort. An appropriate procedure for calculating *Y* is the unbiased estimator of true species richness, the so called Mao Tau estimator (hereafter STE) [41], [61]. Another way of estimating the slope of the SAC is to calculate the species accumulation rate at a given sampling level, by fitting a function to the curve. To do that, it is necessary to previously examine the level of homogeneity of sample units by comparing the empirical mean randomized SAC, with the expected curve if all individuals had been randomly assigned to the samples. The expected curve may be constructed either by computing a rarefaction curve or a Coleman curve (for details see [40], [46]). The slope of the SAC is then calculated with the first derivative of the fitted curve. The final slope of the Clench function (as well as the slope of other asymptotic functions) is the most common method for assessing the accumulation rate [30], [39], [60]. Two main problems are associated with these asymptotic functions: their limited use at low sampling levels of sampling [15], and the violation of statistical assumptions inherent to non-linear regression models (i.e., correct mean structure, variance homogeneity, and independent and normally distributed errors [62]).

### FIDEGAM: a new method to quantify sampling completeness

As an alternative to the methods reviewed above, we have developed the FIDEGAM method, which fits a Generalized Additive Model (GAM) [63]–[64] with Poisson response, or the negative binomial if data presents overdispersion [65], to each randomized SACs. GAM is an extension of Generalized Linear Models (GLM) [66], which allows flexible modeling of the influence of the response variable [64]. In a GAM framework, statistical assumptions are met because the function is adjusted to non-normal distributed data instead of forcing data to fit an arbitrary known function. Besides, contrary to asymptotic methods the model fits even at low levels of sampling effort. Once GAMs are fitted, the first derivatives and their 95% corresponding confidence intervals along the species accumulation process are calculated. This output describes the whole pattern of species accumulation, being the first derivative at the maximum number of sampling records the measure of sampling completeness (onwards FIDEGAM value). FIDEGAM values range from 0 to 1, corresponding to high and low sampling completeness for a given area, respectively.

### Testing and comparing the discrimination capability of the sampling completeness measures

#### (i) Classification rules to assess discrimination capability

The discrimination capability between different methods should be evaluated under different levels of sampling completeness and according to an objective classification rule [43]. From a statistical point of view, the discrimination capability of a given *Y* (e.g. a measure of sampling completeness in our case) to *d*istinguish between two alternative states *S*1 (e.g. well sampled unit) and *S*2 (e.g. poorly sampled un*i*t), should be based on a Receiv*e*r Operating Characteristics (ROC) curve analysis [43], [67], [68]. A binary response is needed for ROC analyses, so that *Y* classifier should be dichotomized according to a cut-poi*n*t value. Values of the sampling unit above that cut-point would refer to one of the two possible states (i.e. *S*1), and values below to the alternative state (i.e. *S*2). *T*he classification criterion used in ROC analysis is *r*elated to the probabilities of belonging to one of the states as a function of the values of *Y*, *P* [*S*1|*Y* ]. These probabilities are estimated using a GL*M* i*n* a bi*na*ry regression framework [43]. Once the ROC curve for each completeness measure is fitted, their discrimination performance is evaluated using the area under the ROC curve (AUC)
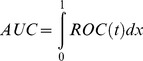
(2)The AUC takes values between 0.5 (uninformative classifier) and 1 (perfect classifier) [44], [69], [70].

#### (ii) Discrimination capability of methods in different scenarios

We tested the discrimination capability of the completeness measures based on the observed proportion of species richness out of the total predicted by a non-parametric estimator (NPE), the slope of the Mao Tau estimator (STE) and FIDEGAM using ROC in two contrasting scenarios of sampling completeness and in a ideal situation, where the true species richness is known.

The scenario of high sampling exhaustiveness is derived from a database that contains information of the vascular flora of the Ordesa-Monte Perdido National Park (Spanish Pyrenees; ORDESA thereafter). The National Park is one of the most exhaustively prospected areas in the Iberian Peninsula [71], however, due to the high topographic complexity, the large altitudinal range (∼700 to 3354 m a.s.l.) and severe access difficulties to some points, the sampling effort is expected to be unevenly distributed along the *ca.* 30000 ha of the Park. The ORDESA database comprises more than 44000 spatially explicit records of 1379 vascular plant species along the 321 UTM cells (1 km^2^; sampling units) of the Park (excluding Bujaruelo valley), compiled from herbarium samples, phytosociological *relevés*, and *visu* records collected over the last 50 years in the JACA Herbarium (http://proyectos.ipe.csic.es/floragon/index.php). To homogenize the different sources of information we defined “sampling record” as each input of information of plants occurrence (from one to multiple species) that differs in date, site, method and/or author.

The second scenario was created emulating the structure (sampling units/sampling records/species per record) of the ORDESA database and using a random procedure, which involves the following steps (see Figure S1 for further details):

1. For each sampling unit (n = 180), assign the number of sampling records according to three levels of sampling intensity (20–50, 51–80 and 81–110 sampling records) at random.
2. For each sampling record, randomly determine how many (between 1 and 30) and which species are recorded from a virtual pool (400 species).

The resultant information was compiled in a database named SIMULAU. We assumed that the sampling effort has been enough to detect the true richness in all the sampling unit of this database. We then subsampled from SIMULAU to achieve an scenario of low sampling exhaustiveness (SIMULAU_sub_). To do so, we repeated Steps 1 and 2, but in this case the number of sampling records and species was randomly assigned according to the information gathered in SIMULAU. To ensure low levels of sampling exhaustiveness in SIMULAU_sub_, we limited the maximum number of sampling record per sampling unit, and the maximum number of detected species per sampling record to 25 and 20, respectively.

**Figure 1:**
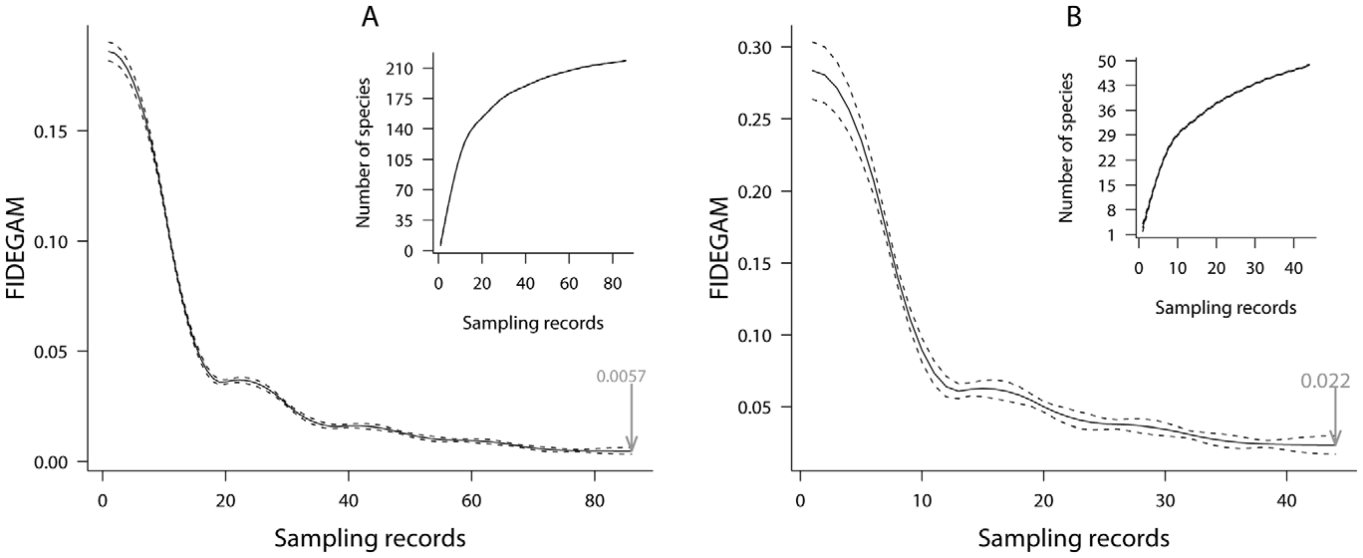
The sampling completeness measured from two smoothed species accumulation curves (1000 randomization each) using the FIDEGAM method in the Ordesa-Monte Perdido National Park. FIDEGAM values (in grey) recorded at the maximum number of sampling records indicates higher sampling completeness in the sampling unit A than in B. Dashed lines correspond to confidence intervals of FIDEGAM values.

The next step was to produce the smoothed SAC for each sampling unit in the ORDESA and SIMULAU_sub_ database using *specaccum* function (1000 permutations) in the VEGAN package [72] in R [73]. Then, the three completeness measures were estimated for each SAC. The NPE was calculated as the proportion of species richness out of the total predicted by the Jacknife estimator using the *poolaccum* function (1000 permutations) in the VEGAN package. The slope for Mao Tau estimator (STE) was computed from the SACs as
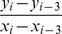
(3)being *i* the last position of both species richness (*y*) and number of records (*x*). We finally used FIDEGAM method by fitting GAM models with Poisson response to the each accumulation curve (obtained at random) using penalized splines [64], [74]. Optimum effective degrees of freedom (equivalents to degrees of smoothness) were automatically selected using the unbiased risk estimator criterion (UBRE) [75]. The first derivative of the resultant curve and its 95% confidence intervals were computed for each sampling unit (Figure 1), being the first derivative at the maximum number of sampling records the FIDEGAM measure of sampling completeness (Figure 1).

The final step consisted in examining the discrimination performance of the three completeness measures calculated, to correctly classify well and poorly sampled sampling units. In most real situations, the true species richness is unknown, therefore, a surrogate of the sampling effort is needed to categorize sampling units. Here, we used the number of sampling records as a surrogate in the ORDESA and SIMULAU_sub_ [30], [47], [76]. We set the cut-point value according to the preliminary analysis [77] at the 50^th^ percentile (i.e. the median) of the number of records per sampling unit [78] (see further details on Appendix S1). Thereby, units with higher number of sampling records than the median were classified as well sampled and those below as poorly sampled. To evaluate the role that the surrogate could play on the results, we categorize the sampling units of SIMULAU according to an ideal scenario where the degree of sampling completeness is known. Given that all species were detected in the sampling units of SIMULAU, we calculated the true sampling completeness for each unit as the
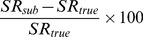
(4)where *SR_sub_* is the species richness in SIMULAU_sub_ and *SR_true_* the true richness obtained from the SIMULAU database [79]–[80]. The inventory of sampling unit exceeding the 70% of completeness are usually considered as nearly completed [81]–[82], therefore, we categorized sampling units according to such value.

Once the binary response variable was created for all scenarios, we proceed to estimate the capability of each completeness measure (NPE, STE and FIDEGAM) for discriminating between classes using ROC-GLM regression for binary responses with logit link. The probability of belonging to each state was calculated as a function of the value of completeness measure
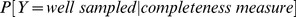
(5)whereas AUC values were computed using the *roccurve* function in the *pcvsuite* package [83] and their 95% confidence intervals estimated by bootstrap regression techniques [84].

## Results

**Figure 2:**
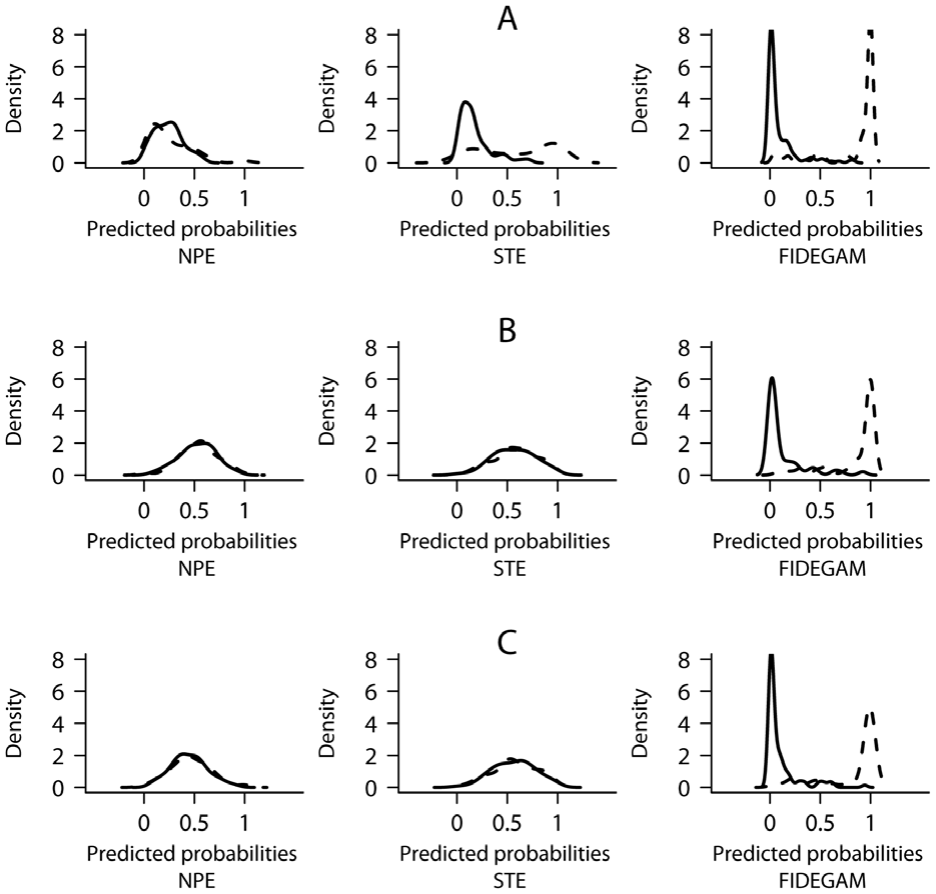
Kernel density plots of predicted probabilities of discrimination between well (dashed line) and poorly sampled units (continuous line) for NPE, STE and FIDEGAM methods. In the scenarios of high (A) and low (B) levels of sampling exhaustiveness, the sampling units were categorized as well and poorly sampled according to the number of records (see Appendix S1), whereas, when the true richness was known (C), the true sampling completeness (see equation 4 on text) was used as a categorization criterion. Probabilities were calculated according to ROC-GLM regression models.

According to our logistic model (equation 5), the predicted probabilities of a method for correct discrimination should reach 1 and 0 for well and poorly sampled units, respectively. Therefore, when representing this ideal discrimination in a kernel density plot, maximum densities of predicted probabilities of well and poorly sampled areas should clump at 0 and 1 values of the x-axis. On the contrary, higher densities of predicted probability values would lie between 0 and 1 if the method fails in discriminating. Figure 2 shows strong differences in the predicted probabilities for well and poorly sampled units among methods, evidencing the higher discrimination capacity of FIDEGAM. This pattern was consistent in the three examined scenarios despite that different surrogates for categorizing the sampling units were used if true richness was known or unknown (Figure 2). In all cases, FIDEGAM showed an excellent performance for discrimination according to the observed AUC values (Table 1). On the contrary, NPE failed to correctly discriminate sampling units in the ORDESA database, whereas, STE only classified correctly poorly sampled units (Figure 2A), being good the discrimination quality (Table 1).

**Table 1:** AUC values and 95% bootstrap confidence intervals (in brackets) obtained in the discriminatory analysis of methods for sampling completeness quantification.

Method	Level of sampling exhaustiveness		SR_true_
	high	low	
NPE	0.64 (0.59, 0.71)	0.49 (0,41, 0.59)	0.52 (0.40, 0.59)
STE	0.81 (0.75, 0.86)	0.49 (0.40, 0.57)	0.48 (0.40, 0.57)
FIDEGAM	0.92 (0.88, 0.95)	0.98 (0.97, 1.00)	0.97 (0.95, 0.98)

NPE is the proportion of species richness out of the total predicted by a non-parametric estimator (Jacknife) [57]; STE is Mao Tau estimator [41], [61]; FIDEGAM is the first derivate of a GAM with Poisson response fitted to species accumulation curves.

The analyses were repeated in two scenarios of high and low levels of sampling exhaustiveness (from the ORDESA and SIMULAU_sub_ databases, respectively), and in an ideal situation where the true species richness was known (SR_true_). Grading guidelines for AUC values indicate fail (0.50–0.60), poor (0.60–0.70), fair (0.70–0.80), good (0.80–0.90) and excellent (0.90–1.00) discrimination.

At lower levels of sampling exhaustiveness (i.e., using data from SIMULAU_sub_), the probability of NPE and STE for correct discrimination decreased (Figure 2B), reaching undesirable AUC values (Table 1). The same results were obtained in the simulated scenario when sampling units were categorized according to the true sampling completeness (Figure 2C, Table 1).

**Figure 3:**
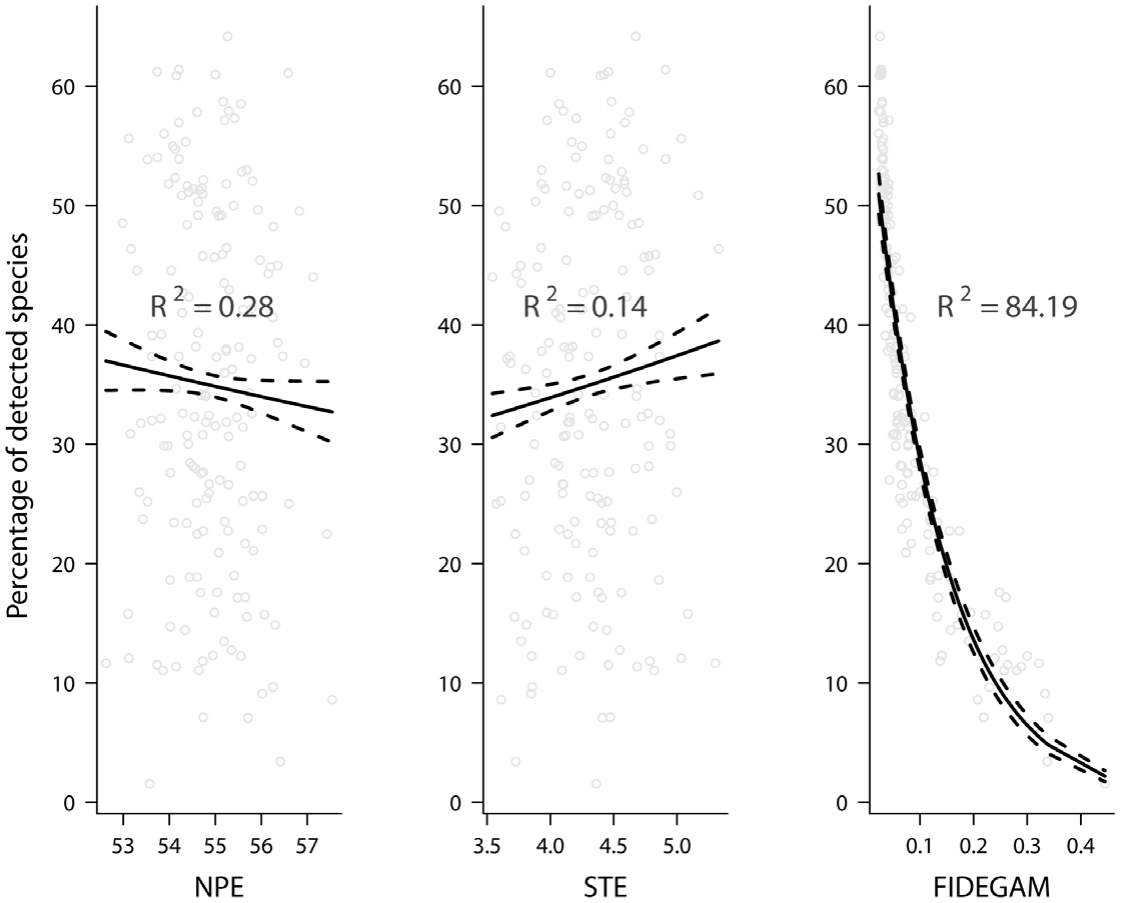
Relationship between the sampling completeness calculated using the FIDEGAM method and the percentage of detected species in a simulated scenario, where the true richness is known. Dashed lines state the 95% confidence intervals.

To better interpret the results obtained in the discrimination analysis, we plotted the relationship between the true percentage of species detected (defined as the ratio between the richness observed in SIMULAU_sub_ and SIMULAU) and the completeness values of NPE, STE and FIDEGAM in SIMULAU_sub_ (Figure 3). By fitting a Poisson-GLM to this relationship, we found that values of both NPE and STE did not correlate with that (R^2^ = 0.28 and R^2^ = 0.14, respectively), whereas FIDEGAM values did (R^2^ = 84.19) (Figure 3).

### Handling with sampling effort bias in biodiversity analyses: a case study

To illustrate how the measure of sampling completeness can be used to enhance the reliability of biodiversity analysis, we analyzed the patterns of distinctiveness along the Ordesa-Monte Perdido National Park (excluding Bujaruelo valley) using the ORDESA database. The distinctiveness indicates to what extent one area is distinct from other areas in terms of taxonomic, functional or/and genetic diversity [85]–[86]. The identification of most distinctive areas constitutes a basis for establishing priority conservation areas at different scales. We calculated here an easy-to-use index based on taxonomic distinctiveness according to the formula detailed in Jennings et al. [81], but it is also possible to use other metrics of distinctiveness and beta diversity.

**Figure 4:**
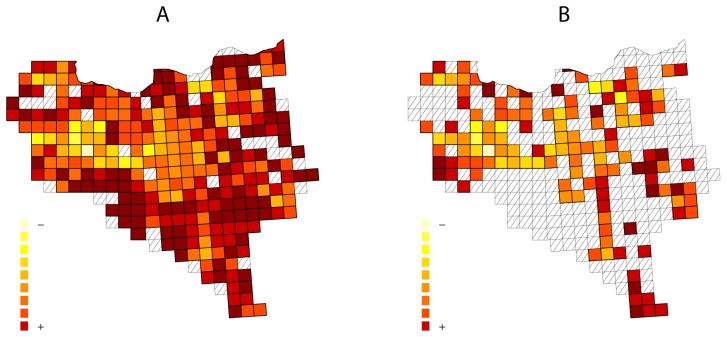
Taxonomic distinctiveness in the Ordesa-Monte Perdido National Park calculated using all (A) and selected (B) sampling units. Grid cells correspond to UTMs of 1 km^2^. Striped cells indicates sampling units with less than three sampling records, where the quantification of sampling completeness is impossible using FIDEGAM method, in A, and poorly sampled units in B. Well and poorly sampled units were defined using their completeness value of FIDEGAM and according to a threshold value that maximize the discrimination capability between sampling units (see text for details).

**Figure 5:**
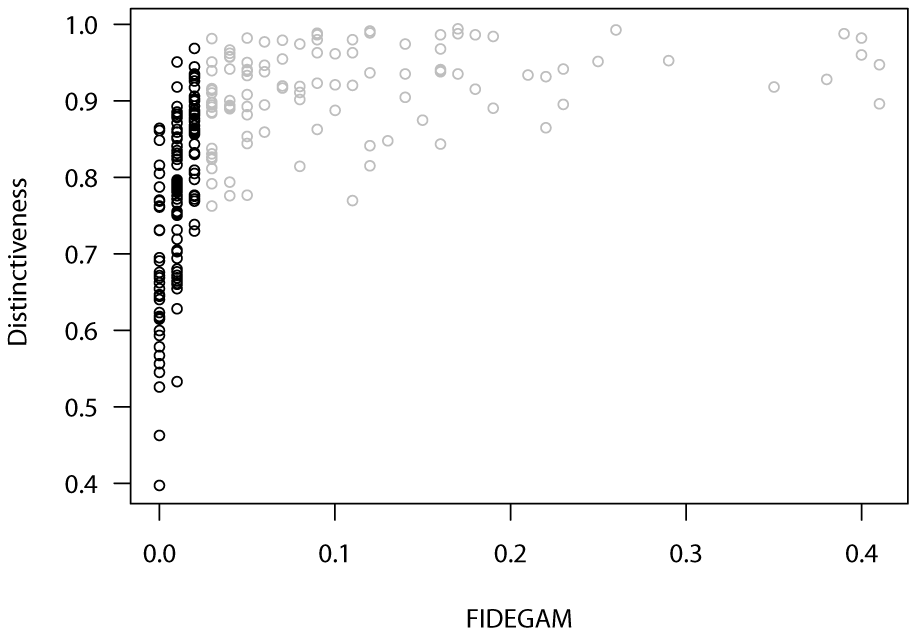
FIDEGAM values and taxonomic distinctiveness in the Ordesa-Monte Perdido National Park. Low values of FIDEGAM correspond to high sampling completeness. Black and grey dots indicate well and poorly sampled units (1 km^2^) respectively, according to an optimum threshold value of FIDEGAM that maximizes discrimination capability.

Figure 4A represents distinctiveness in the National Park from the raw information in ORDESA, and suggests that most areas of the Park would be highly distinctive. To what extent is this pattern reliable? We quantified the sampling completeness of each sampling unit with FIDEGAM and found that most of the poorly sampled ones were those of highest distinctiveness values (Figures 4 and 5). Hence, the distinctiveness pattern obtained from raw information is highly uncertain. To minimize such uncertainty, we excluded poorly sampled areas from analysis according to an objective criterion based on the maximization of the discrimination capability using the Youden index (*J*) [87]. The *J* value in the ROC curve is
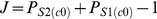
(6)being *P* the probability of correctly classifying, *S*1 and *S*2 well and poorly sampled units respectively, and *c_0_* the optimum cut-point, and the corresponding value of FIDEGAM the optimum threshold to separate well sampled units from poorly. In the ORDESA database the *J* index was 0.85 (confidence interval: 0.75–0.93) and the corresponding threshold 0.029. After excluding sampling units with FIDEGAM values above such threshold (i.e., poorly sampled areas), we recalculated the distinctiveness values and found that the resulting pattern of distinctiveness totally differed from the previous one (Figure 4B). This result evidences how the inclusion of uncertain information in biodiversity analysis (poorly sampled units in this case) distorts the overall picture of the spatial pattern of distinctiveness.

## Discussion

Many biodiversity databases have been constructed from heterogeneous sources of information because of the large spatio-temporal ranges they usually cover. The information that they contain, therefore, does not always represent the reality due to large differences in sampling effort across time and space. This fact constitutes one of the main limiting factors for the reliability of the results provided by analyses based on them. Different methods have been proposed to account for spatial sampling effort bias, but not all of them perform equally. Here, we have demonstrated that SAC based methods differ in terms of statistical robustness, but also in their capability to discriminate between well and poorly sampled units.

Statistical assumptions cannot be disregarded even in the most-up-date statistical methods [88]. Some methods for quantifying sampling completeness do not fulfill such assumptions (see [89]), whereas others (e.g. the classic asymptotic function [39]) present severe limitations. Even when the statistical assumptions are not violated, not all methods are equally reliable because there are strong differences in their discrimination capability, as we have shown here. The novel method we have proposed in this study, FIDEGAM, outperforms others regardless of the sampling exhaustiveness, and both when true richness was known and unknown, evidencing its robustness. The most striking feature of the method is its excellent performance at low levels of exhaustiveness, because most regions and living groups worldwide are not exhaustively sampled [15]. In turn, other methods based on NPE and STE estimators, often misclassified well and poorly sampled units, which may constitute another source of bias to the original problem of sampling effort bias. As a result of this low discrimination capacity, NPE and STE also failed to represent the true proportion of detected species in a simulated scenario.

The sampling exhaustiveness of the database is an important constrain for the use of both non-parametric estimators and asymptotic methods [90]. Beyond discrimination capability, even the computation of sampling completeness values is limited by using asymptotic methods (e.g. Michaelis-Menten) in scarcely prospected areas. These areas ought to be ruled from the sampling effort assessment, and as a result, a large amount of information is susceptible to be lost. This situation is less dramatic using the FIDEGAM method, because it requires a lower number of sampling records (i.e. three) than the asymptotic ones.

The assessment of sampling completeness can be easily incorporated into biodiversity analyses to reduce the uncertainty of results. A promising procedure is to incorporate sampling completeness values as a covariate (or offset) in the analysis of biodiversity patterns (Pata et al., unpublished data), although the most frequent alternative is to only consider the areas that are well surveyed (i.e. those with a sampling effort above some threshold) [15], [91], [92], [93]. If sampling effort is similarly distributed across space (regardless of the level of sampling exhaustiveness), the selection should be done according to comparable values of sampling effort rather than to high values [35], [94]. The full interest of this procedure relies on how to define a threshold value in order to classify the suitability of different areas [30], [95]. An arbitrary value may be justified when the knowledge of the studied system is robust, otherwise the subjectiveness should be avoided. In the example presented, the threshold value was defined according to the maximization of discrimination capability, thereby, minimizing in this way the potential bias intrinsic to method. The straightforward advantage of proceeding objectively is that the method can be equally used in other databases, regardless of the nature and spatial resolution of the information.

Correctly identifying well and poorly sampled areas is also of paramount importance for the interpretation of biodiversity distribution [27], [92], and FIDEGAM has been proved to provide an accurate layer of uncertainty over results obtained from raw data. This would allow us to know at which locations results of biodiversity analysis is reliable, and where the prospective biological exploration is necessary if we want to extend results of standard analysis of biodiversity [53], [92].

To summarize, our results have highlighted that an adequate selection of the assessment method is as important as the decision itself of taking into account the sampling effort for enhancing the reliability of database analyses. FIDEGAM provides the best discrimination capability and minor dependence on exhaustiveness. Therefore, we recommend this method to overcome sampling effort bias when analyzing the information gathered in biodiversity databases. By no means, a method for sampling completeness quantification will replace the advantages provided by further biological prospections. However, given the urgencies of biodiversity conservation and the limitations for intensive data gathering, we consider the quantification of sampling completeness the best alternative to enhance the reliability of biodiversity analyses based on non-exhaustive database.

## Supporting Information

Figure S1 **Diagram of the simulation procedure to create the ideal scenario, where the true richness is known (SIMULAU), and the scenario of low levels of sampling exhaustiveness (SIMULAU_sub_).**
(PDF)

Appendix S1 **Preliminary studies to establish an adequate threshold based on the number of sampling records for the discrimination analysis.**
(PDF)
