# Supplementary material for: A Novel Method to Handle the Effect of Uneven Sampling Effort in Biodiversity Databases
Source: PLoS One. 2013 Jan 11;8(1):e52786. doi: 10.1371/journal.pone.0052786 (PMC3543413; doi:10.1371/journal.pone.0052786)

Figure S1. Diagram of the simulation procedure to create the ideal scenario, where the true richness is known (SIMULAU), and the scenario of low levels of sampling exhaustiveness ($SIMULAU_{sub}$). Dashed lines state a random assignation of the number of sampling records to a sampling unit (a; a'), the number of species detected in a given sampling records (b; b'). In the SIMULAU database the total number of species observed at each of the sampling units was defined as the true richness. We generated the scenario of low sampling exhaustiveness ($SIMULAU_{sub}$) by subsampling at random the SIMULAU database. Note that we restricted the number of record per sampling unit and the maximum number of species observed per record, to ensure low levels of sampling completeness.

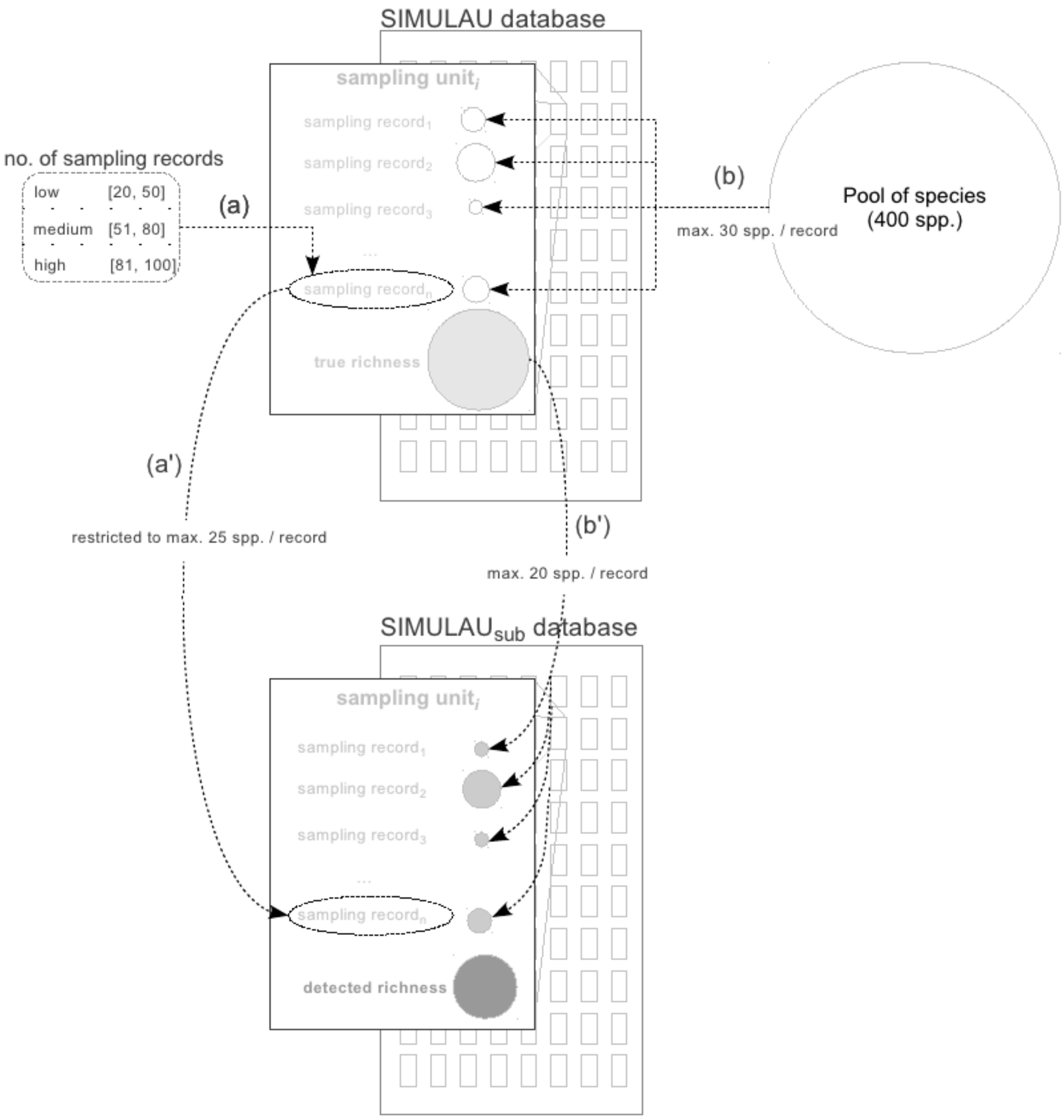

Supplement: Figure S1 — Diagram of the simulation procedure to create the ideal scenario, where the true richness is known (SIMULAU), and the scenario of low levels of sampling exhaustiveness (SIMULAUsub). (PDF) [file pone.0052786.s001.pdf]
