# Supplementary material for: A Novel Method to Handle the Effect of Uneven Sampling Effort in Biodiversity Databases
Source: PLoS One. 2013 Jan 11;8(1):e52786. doi: 10.1371/journal.pone.0052786 (PMC3543413; doi:10.1371/journal.pone.0052786)
Supplement: Appendix S1 — Preliminary studies to establish an adequate threshold based on the number of sampling records for the discrimination analysis. (PDF) [file pone.0052786.s002.pdf]

Appendix S1. Preliminary studies to establish an adequate threshold based on the number of sampling records for the discrimination analysis.

The establishment of a threshold to separate two states (e.g. well and poorly sampled) was done using graphical diagnostic and model-based plots, and, in the case of the SIMULAU database, using the maximum chi-square method [1- 3].

In the simulated scenario where the true richness was known (SIMULAU), we could easily transform the richness into a binary variable according to a common accuracy measure [4-6] as

$$\frac{SR_{sub} - SR_{true}}{SR_{true}}$$

where SR_{sub} is the species richness subsampled from the simulated scenario ($SIMULAU_{sub}$) and SR_{true} the richness observed in each of the sampling unit in SIMULAU. The inventory is considered nearly complete above 70% of such measure [7-8 but see 9], therefore, we defined well sampled units as those exceeding such values. Once the binary variable was obtained, we calculated the proportion of poorly sampled units in SIMULAU to the mean number of records by quantile (Figure A1).

The Figure A1 confirms the existence of a threshold: sampling units with more sampling records than the median were well sampled, whereas, those with less number were poorly sampled. The difference between well and poorly sampled units is ca. 50% as separated by median (Figure A1). Additionally, we modeled the probability of units for being poorly sampled as a function of number of sampling records using a binomial GLM, and found consistent results (Figure A1). Please, note that the same conclusion (but inverse results) would be achieved in these analyses using the well sampling units.

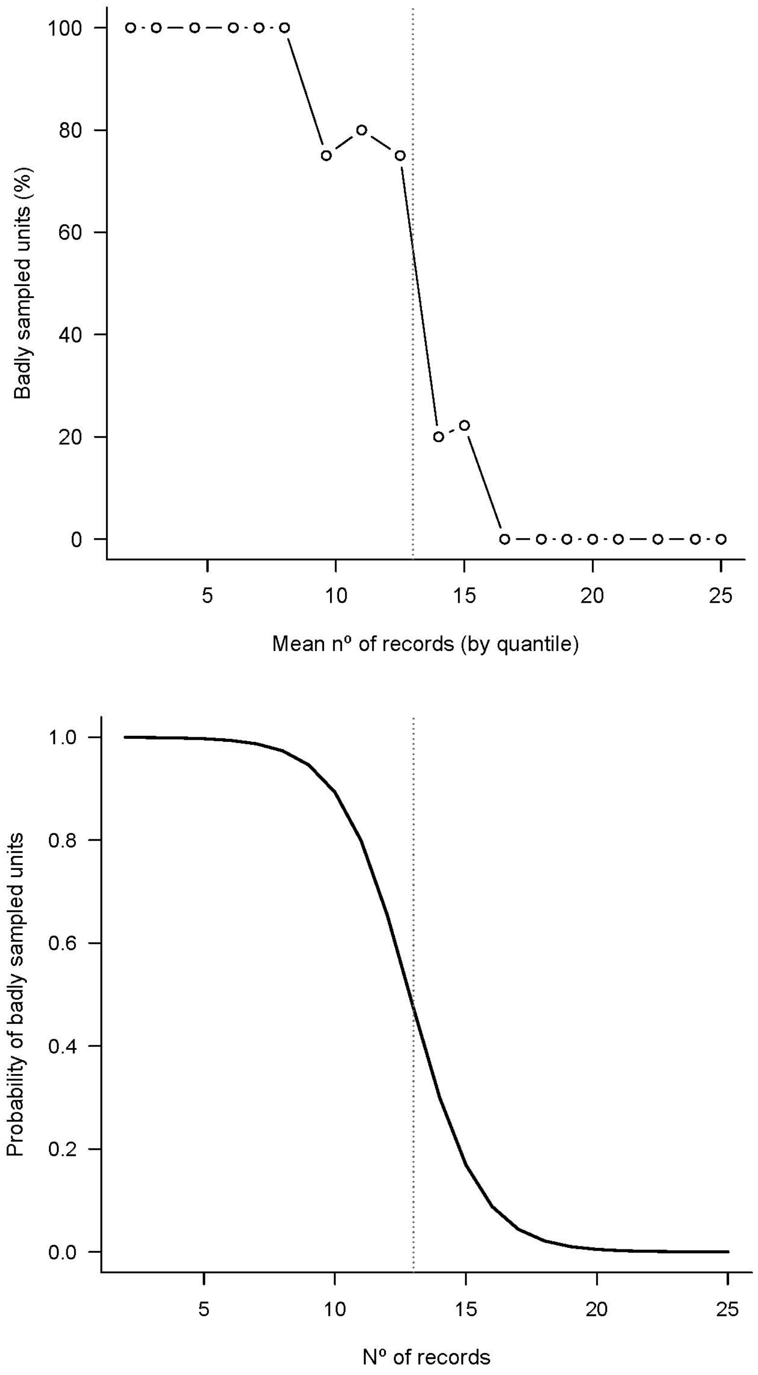

Figure A1. Percentage of poorly sampled units to the mean number of sampling records by quantile (top), and binomial GLM-based plot of the probabilities of poorly sampling units as a function of the number of sampling records (bottom) in a simulated scenario. Dotted line states the median of the number of sampling records.

Finally, we tested for the best threshold value, using the maximum chi-square method. In this method the quantiles of the number of sampling records are evaluated as a cut-points (i.e.threshold). A two-sampled test with the corresponding chi-square statistic and p-value was computed for each quantile, and that with a maximum chi-square statistic (or equivalently the minimum p-value) was selected as the cut-point which best separates well and poorly sampled units. We corrected the p-values to avoid type I errors due to multiple testing [1]. We found that the median of the number of sampling records yields the highest statistic and the lowest p-value (Table A1 and Figure A2).

Table A1. Results of the maximum chi-square test to find the best threshold to separate sampling units. The test was repeated for each quantile, and the maximum and minimum chi-square statistic and p-value (in bold), respectively, indicates the number of sampling records which best separate well and poorly sampling units .

Quantile	Cut-point	Chi-square	p-value
5	2,95	7,49	6,22E-003
10	3,90	17,84	2,40E-005
15	5,00	33,64	6,63E-009
20	6,00	44,09	3,14E-011
25	7,75	57,36	3,62E-014
30	8,00	76,35	2,38E-018
35	10,00	89,41	3,21E-021
40	11,00	103,79	2,25E-024
45	11,55	103,79	2,25E-024
**50**	**13,00**	**129,14**	**6,33E-030**
55	14,00	120,57	4,76E-028
60	15,00	108,67	1,91E-025
65	17,00	91,55	1,09E-021
70	18,30	74,31	6,67E-018
75	19,00	53,90	2,11E-013
80	20,00	38,00	7,09E-010
85	21,00	28,10	1,15E-007
90	23,00	11,94	5,50E-004
95	24,00	6,41	1,13E-002

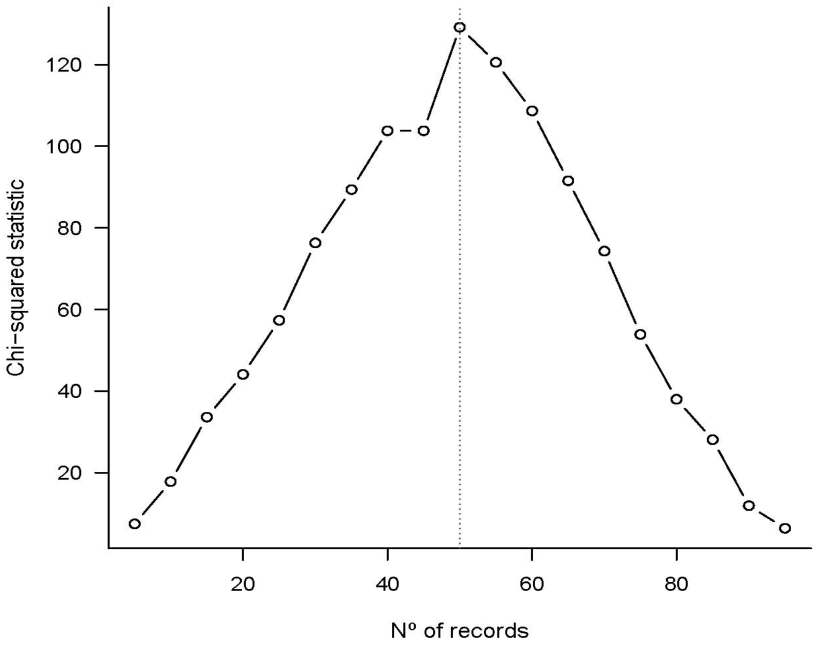

Figure A2. The relationship between the number of sampling records and statistics obtained using the maximum chi-square test. Dotted line states the median of the number of sampling records.

Given that the true richness is unknown in the real scenario built from the ORDESA database, we cannot applied the previous procedure. As an alternative, we modeled the relationship between the number of sampling records and observed richness using two approaches:

(a) Piecewise regression, which is based on regression models that joins two or more lines at some unknown points (breakpoints) [10-11]. The resulting breakpoints can be considered as the thresholds for the variable in study (sampling records in our case).

(b) Quantile regression, which estimates the relationships between richness (response variable) and number of sampling records (covariate) for all quantiles of the distribution [12-13].

The unique breakpoint resulting from the piecewise regression was 32.6 (± 0.618 standard error), which corresponds to the 55th quantile. Figure A3 shows the fitted lines obtained with the piecewise regression model. In line with this result, we found different estimated slopes (i.e. different relation between species richness and number of sampling records) above and below the median using the quantile regression (Figure A4).

Altogether, the results obtained in the simulated and the ORDESA databases support that the median of the number of sampling records was the most adequate threshold value for the discrimination analysis.

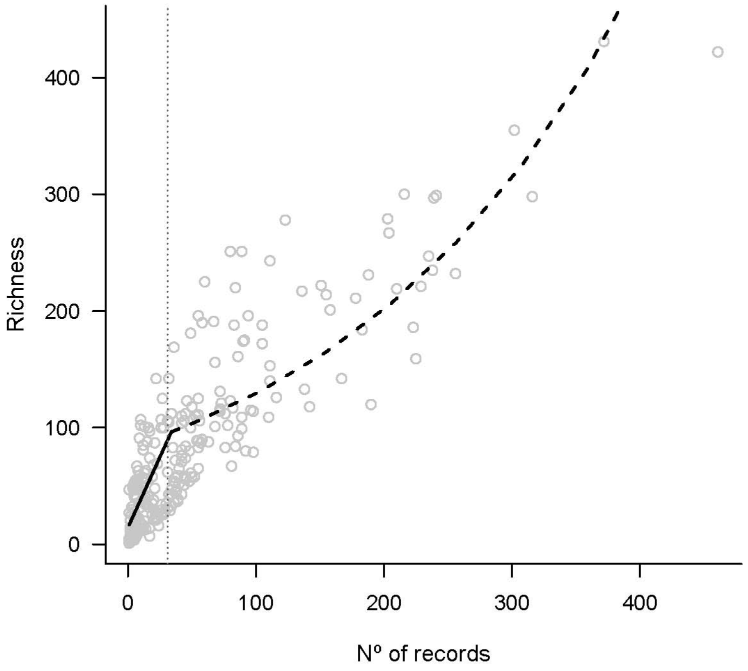

Figure A3. Piecewise regression of the sampling records and the species richness. Lines represent the fitted models above (dashed line) and below (black line) the obtained breakpoint, which matchs with the median of the number of sampling records (dotted grey line).

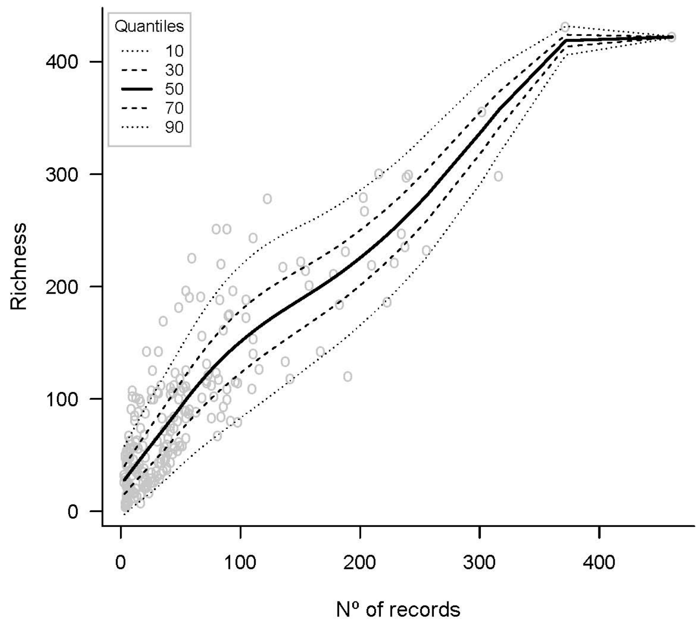

Figure A4. Scatter plot of number of sampling records and species richness. Black lines represent the estimated slopes obtained in a quantile regression.

References

1. Miller R, Siegmund D (1982) Maximally selected chi squared statistics. *Biometrics* 38: 1011−1016.

2. Lausen B, Schumacher M (1992) Maximally selected rank statistics. *Biometrics* 48: 73−85.

3. Mazumdar M, Glassman JR (2000) Categorizing a prognostic variable: review of methods, code for easy implementation and applications to decision-making about cancer treatments. Stat. Med. 19: 113−132.

4. Chiarucci A, Maccherini S, De Dominicis V (2001) Evaluation and monitoring of the flora in a nature reserve by estimation methods. Biol. Conserv. 101: 305–314.

5. Brose U (2002) Estimating species richness of pitfall catches by non-parametric estimators. Pedobiologia, 46: 101−107.

6. Walther A, Moore JL (2005) The concepts of bias, precision and accuracy, and their use in testing the performance of species richness estimators, with a literature review of estimator performance. Ecography, 28: 815−829.

7. Williams VL, Witkowsky ETF, Balkill K (2007) The use of incidence-based species richness estimators, species accumulation curves and similarity measures to appraise ethnobotanical inventories from South Africa. Biodivers. Conserv. 16: 2495−2513.

8. Mora C, Tittensor DP and Ransom A Myers RA (2008). The completeness of taxonomic Inventories for describing the global diversity and distribution of marine fishes. Proc. R. Soc. B 275: 149–155.

9. Lobo JM (2008) Database records as a surrogate for sampling effort provide higher species richness estimations. Biodivers. Conserv. 17: 873–881.

10. Muggeo V (2003) Estimating regression models with unknown break-points. Stat. Med. 22: 3055−3071.

11. Toms JD, Lesperace ML (2003) Piecewise regression: a tool for identifying ecological thresholds. Ecology 84: 2034−2041.

12. Cade BS, Terrell JW, Schroeder R (1999) Estimating effects of limiting factors with quantile regression. Ecology 80: 311−323.

13. Koenker R, Pin Ng, Portnoy S (1994) Quantile Smoothing splines. Biometrika, 81 (4): 673−680.
